# Cigarette pack size and consumption: an adaptive randomised controlled trial

**DOI:** 10.1186/s12889-021-11413-4

**Published:** 2021-07-18

**Authors:** Ilse Lee, Anna K. M. Blackwell, Michelle Scollo, Katie De-loyde, Richard W. Morris, Mark A. Pilling, Gareth J. Hollands, Melanie Wakefield, Marcus R. Munafò, Theresa M. Marteau

**Affiliations:** 1 Behaviour and Health Research Unit, University of Cambridge, Cambridge, CB2 0SR UK; 2 School of Psychological Science, University of Bristol, 12a Priory Road, Bristol, BS8 1TU UK; 3 Centre for Behavioural Research in Cancer, Cancer Council Victoria 615 St Kilda Rd, Melbourne, Vic 3004 Australia; 4 Department of Population Health Sciences, Bristol Medical School, University of Bristol, Bristol, UK

**Keywords:** Tobacco control, Adaptive design, Cigarette packaging, Pack size

## Abstract

**Background:**

Observational evidence suggests that cigarette pack size – the number of cigarettes in a single pack – is associated with consumption but experimental evidence of a causal relationship is lacking. The tobacco industry is introducing increasingly large packs, in the absence of maximum cigarette pack size regulation. In Australia, the minimum pack size is 20 but packs of up to 50 cigarettes are available. We aimed to estimate the impact on smoking of reducing cigarette pack sizes from ≥25 to 20 cigarettes per pack.

**Method:**

A two-stage adaptive parallel group RCT in which Australian smokers who usually purchase packs containing ≥25 cigarettes were randomised to use only packs containing either 20 (intervention) or their usual packs (control) for four weeks. The primary outcome, the average number of cigarettes smoked per day, was measured through collecting all finished cigarette packs, labelled with the number of cigarettes participants smoked. An interim sample size re-estimation was used to evaluate the possibility of detecting a meaningful difference in the primary outcome.

**Results:**

The interim analysis, conducted when 124 participants had been randomised, suggested 1122 additional participants needed to be randomised for sufficient power to detect a meaningful effect. This exceeded pre-specified criteria for feasible recruitment, and data collection was terminated accordingly. Analysis of complete data (*n* = 79) indicated that the mean cigarettes smoked per day was 15.9 (SD = 8.5) in the intervention arm and 16.8 (SD = 6.7) among controls (difference − 0.9: 95%CI = − 4.3, 2.6).

**Conclusion:**

It remains unclear whether reducing cigarette pack sizes from ≥25 to 20 cigarettes reduces cigarette consumption. Importantly, the results of this study provide no evidence that capping cigarette pack sizes would be ineffective at reducing smoking. The limitations identified in this study can inform a more efficient RCT, which is urgently required to address the dearth of experimental evidence on the impact of large cigarette pack sizes on smoking.

**Trial registration:**

10.1186/ISRCTN34202533

**Supplementary Information:**

The online version contains supplementary material available at 10.1186/s12889-021-11413-4.

## Background

Despite progress in global tobacco control [1], smoking remains one of the largest risk factors for disease globally [2] and a major cause of the gap in healthy life expectancy between the richest and poorest [3]. It has been argued that cigarette pack size – the number of cigarettes in a single pack –should be subjected to increased regulation [4].

There is variation between countries in the range of pack sizes available. A minimum of 20 cigarettes per pack has been imposed by many countries (e.g., the European Union Tobacco Products Directive (2014/40/EU)) in order to maintain the upfront cost of cigarettes, particularly targeting affordability for young people [5]. However, the tobacco industry is introducing larger pack sizes to the market in response to new tobacco control policies [5–7]. In Australia for example, packs of 20, 21, 22, 23, 25, 26, 30, 35, 40, 43 and 50 are all currently available.

Robust experimental evidence suggests that larger portions, packages and tableware increase food consumption [8]. Cigarette pack size is also associated with numbers of cigarettes smoked. A large survey of Australian smokers found that self-reported cigarettes per day was positively associated with pack size [9]. Small packs are used by some smokers as a method of self-regulating consumption [10]. Tobacco industry documents reveal that cigarette brands were released in packs of 25 rather than 20 in an effort to reverse declines in sales by increasing daily consumption [11]. However, experimental evidence for a causal relationship between pack size and consumption is lacking.

If larger packs increase smoking, then introducing a cap on cigarette pack size could be an effective tobacco control measure to reduce smoking and associated health harms. There is broadly, if not exactly, a linear relationship between number of cigarettes smoked and harm caused by smoking [12, 13]. Smoking fewer cigarettes per day increases the likelihood of quit attempts [14] and eventual cessation [15], and is recommended in the UK harm reduction guidelines issued by the National Institute for Health and Care Excellence [16]. A Mendelian randomisation study suggested that smoking one fewer cigarette per day increases the odds of cessation by 9% [17]. The impact of reducing smoking at a population level can be estimated by conservatively assuming a 5% increase in the odds of cessation for each fewer cigarette smoked per day. In Australia for example, a reduction of two cigarettes per day is estimated to reduce smoking prevalence by 0.3% over one year and thereby increase the number of ex-smokers by 6367 a year [4]. Although the size of this policy impact may not be maintained beyond its initial introduction, regulation that prevents the introduction of larger pack sizes could still play an important role in maintaining global declines in smoking prevalence by preventing smokers from switching to larger pack sizes (if this is an outcome which discourages smoking cessation).

In sum, the direct influence of cigarette pack size on smoking while plausible and potentially consequential is currently uncertain. Our study aimed to estimate the impact on cigarette consumption of a policy that caps cigarette pack sizes at 20 in jurisdictions where this is currently also the minimum pack size. We did this by asking Australian smokers who usually smoke from cigarette pack sizes of ≥25 cigarettes to smoke from packs of 20 cigarettes.

We hypothesised that smokers using packs of 20 cigarettes would smoke fewer cigarettes per day than would smokers using packs of ≥25 cigarettes. A pilot study – described in Additional File 1 – was conducted to inform key parameters of the present two-stage adaptive randomised controlled trial (RCT).

## Methods

### Design

This was a two-stage adaptive parallel group RCT with a planned sample size re-estimation conducted at an interim stage of data collection, also known as an internal pilot design. This design was selected because the estimate of within-arm standard deviation (SD) from the external pilot study had a wide confidence interval and the interim analysis would allow for a more accurate estimation of the SD as a basis for assessing whether the expected effect size could be feasibly demonstrated. Adaptive trials can make the most efficient use of resources [18] by potentially allowing conclusions to be reached more quickly, and requiring smaller sample sizes on average than traditional trial designs, or avoiding unnecessary use of resources conducting underpowered trials.

A pre-specified criterion was established to terminate the trial if the sample size re-estimation indicated that more than 250 participants would need to be randomised in order to detect the expected effect. Randomising more than 250 participants was not considered feasible within the available resources.

Appendix 1 provides further information on design decisions and accessing trial registration documents.

### Intervention

Participants were randomised to one of two study arms. Participants in the control arm were instructed to continue smoking their usual brand variant of cigarettes in their usual pack size (25 cigarettes or more). Participants in the intervention arm were instructed to purchase their usual brand variant of cigarettes in pack sizes of 20 cigarettes only. Data collection took place over a period of four weeks.

### Setting

The study took place in Australia, nationwide; data were collected via telephone and post.

### Sample

Australian smokers who smoke at least five cigarettes per day and typically purchase pack sizes of ≥25 cigarettes (see Appendix 2 for all inclusion and exclusion criteria) were recruited by a research agency in Melbourne (Roy Morgan: https://www.roymorgan.com). Data collection took place between October 2018 and January 2019.

Participants were remunerated up to AUD$240 for their time. Participants allocated to the intervention were also reimbursed for the average additional cost incurred from purchasing the same number of cigarettes in smaller pack sizes than usual.

### Sample size

An initial sample size calculation used the estimate of the within-arm SD of cigarettes per day from a pilot study (5.1 (95% CI [3.7, 8.2]: Additional File 1), which indicated that a sample size of 206 participants would give 80% power at the 5% significance level and a two-sided test to detect a difference of two cigarettes per day. This effect size was selected for importance at a population-level (see Introduction), and plausibility.

Sample size was re-estimated during the interim analysis stage. Participants were recruited in weekly batches; therefore, the analysis was planned for the first week after at least 50 participants had provided outcome data, which was considered sufficient [19, 20].

### Randomisation

A simple randomisation sequence was generated by a senior statistician (RM) not involved with recruitment or data collection. The random number list was given to a researcher at the research agency who was not involved in enrolling participants. Participants’ allocation to condition was concealed until after enrolment and completion of the baseline phase (see below). Participants were blinded to the study hypothesis. The analyst completing the data analysis (KDL) was blinded to allocation.

### Measures

#### Primary outcome

The average number of cigarettes smoked per day. The total number of cigarettes smoked over the four-week study period was measured using information participants provided on labels attached to each cigarette pack they smoked during the study (see Materials below). Participants sent their labelled cigarette packs to the research agency at the end of each week. For each participant, the total number of cigarettes smoked over the four-week study period was divided by 28.

#### Secondary outcomes

Heaviness of smoking, motivation to stop smoking and autonomy over smoking were measured at the end of the study, via a telephone survey, using the following scales:

i. Heaviness of Smoking Index (HSI): a two-item measure of the number of cigarettes smoked per day and time to first cigarette [20].

ii. Motivation to Stop Scale (MTSS): a single-item measure [21] with responses to the question: Which of the following describes you? Responses range from 1, I don’t want to stop smoking to 7, I REALLY want to stop smoking and intend to in the next month.

iii. Autonomy Over Smoking Scale (AUTOS): a 12-item measure of tobacco dependence [22, 23].

#### Baseline measures

Age and gender were recorded at recruitment. Socioeconomic status was measured using the Australian index of Relative Socio-Economic Advantage and Disadvantage (SEIFA, 24) which reflects a combination of education level, income and occupation of respondent. Scores (out of 60) for each of these three categories were combined and summarised in population quintiles ranging from the first quintile– lowest socioeconomic status (SES) – to the fifth – highest SES [24]. The HSI, MTSS and AUTOS were also measured at recruitment.

### Materials

#### Cigarette pack labels

A set of white, green and red adhesive labels (5.5 × 6.5 cm) were provided to participants. They were asked to attach a label to each cigarette pack smoked during the four-week study period.

White labels had space for participants to fill in the following information: date pack started, date pack finished, number of cigarettes smoked from the pack, number of cigarettes given away from the pack, and number of cigarettes smoked from another pack (e.g., given by a friend) during the stated dates. The green labels were for the first cigarette pack used at the beginning of the study and additionally had space for participants to record how many cigarettes were in the pack, which may have already been open. The red labels were for the final cigarette pack used at the end of the study, which may have been unfinished, and contained an additional field for the number of cigarettes that remained.

#### Instruction packs

Printed instructions were posted to participants along with a set of labels, return slips and four pre-paid return envelopes for participants to post their finished cigarette packs at the end of each week.

### Procedure

The study was presented to participants as investigating how cigarette pack size affects the perception of health warnings to avoid participants focusing on their smoking in relation to pack size. Participants were asked to rate health warning effectiveness on the pack labels.

After providing informed consent via an online form, participants completed a one-week baseline phase that aimed to familiarise them with the study procedures. Participants were sent one pre-paid envelope in which they were asked to return date-stamped receipts for cigarette packs purchased over the baseline week.

Participants who successfully completed the baseline week were randomly allocated to one of the two study arms and sent an instruction pack by post. Participants were instructed to smoke their regular brand variant of cigarettes from only i. their usual pack size (≥25), or ii. from pack sizes of 20, according to their allocated study arm for the following four-week study period. Participants attached labels to each cigarette pack they finished, filled in the required information and posted them back to the research agency at the end of each week. In the first week only, participants were also asked to return date-stamped receipts for cigarettes purchased that week. These were used to calculate the reimbursement for participants in the intervention condition in cases where purchasing smaller packs incurred a greater cost per cigarette than usual.

Text and telephone call reminders were sent to participants during the study to maximise adherence to the intervention.

A telephone interview was conducted within two weeks of the research agency’s receipt of the final envelope. The true study aim was disclosed to participants during the final telephone debriefing.

### Data analysis

#### Sample size re-estimation

All analyses were conducted by a senior statistician (MP) and analyst (KDL) who were not involved in data collection and were blinded to allocation. The sample size re-estimation procedure was conducted using R statistical software (v3.4) [25]. The mean and SD were calculated for each study arm along with the difference in means with 95% confidence intervals (CIs) and the precise *p*-value.

The remainder of the analyses were conducted in SPSS 24.

#### Sample characteristics

Descriptive statistics were calculated for demographic and smoking characteristics of participants in each study arm.

#### Primary outcome

A full analysis of all outcome variables was conducted. All imputations for missing data, and assumptions concerning inconsistent responses on cigarette pack labels were made prior to analyses (Additional File 2).

The primary analysis was a modified intention-to-treat analysis in which data from participants were included in the study arm to which they were allocated, excluding participants who did not provide complete data. The comparison of the primary outcome between study arms was made by estimating the difference in means using an independent samples t-test. A secondary analysis of the primary outcome was carried out using analyses of covariance (ANCOVA) adjusting for the pack sizes participants reported typically smoking from at recruitment.

A per-protocol analysis was also conducted for the primary outcome, which included only those participants who adhered to instructions by smoking from cigarettes in the instructed pack size. Participants were deemed to be adherent if at least 90% of the cigarette packs they used during the study were of the correct size (excluding packs already had open at the start of the study). The per-protocol analysis was of interest, given our aim of assessing the actual effect of a policy to introduce a maximum size of cigarette packs rather than just asking people to smoke from smaller packs.

#### Secondary outcomes

Differences between study arms in the means of the secondary outcomes were estimated using ANCOVA where adjustment was made for the same measures taken at recruitment.

## Results

### Sample characteristics

**Fig. 1:**
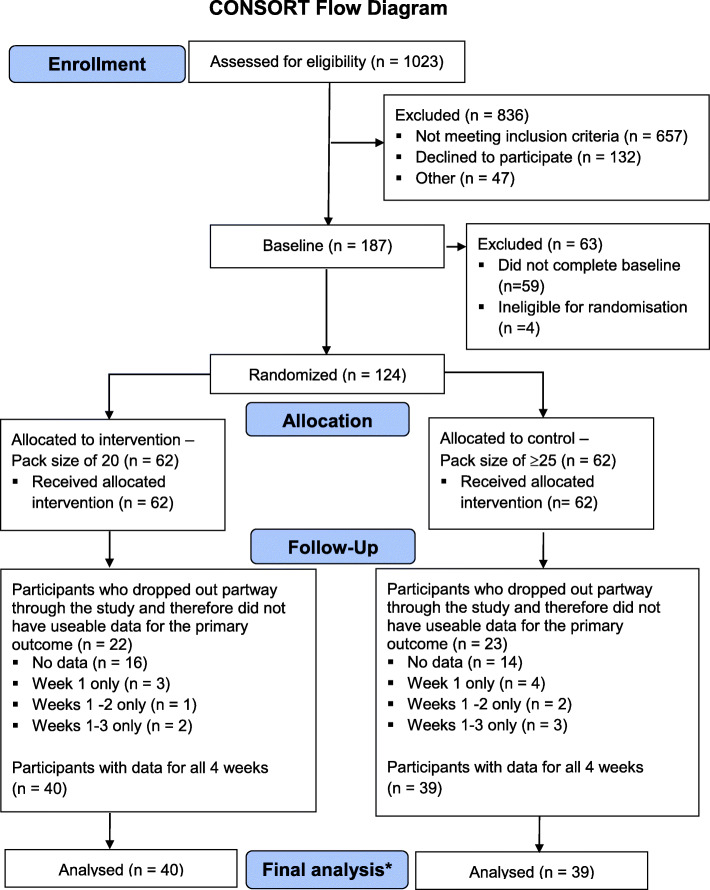
CONSORT flow diagram

Of the 336 smokers meeting the eligibility criteria, 187 (51.1%) consented to take part in the study. 124 participants completed the baseline week and were allocated to one of the study arms (Fig. 1).

Of the 124 randomised participants, 79 (64%) completed the study. All demographic characteristics were comparable between participants who were excluded and those who completed the study (Table 1). The mean age of included participants was 54.6 years (SD = 12.9). Participants were mostly women (57%), had a median education level of 9, indicating they had had some university or college of Advanced Education training, (IQR = 5–12) and mean socioeconomic quintile of 3.1 (SD = 1.3) with 38% of participants in the top two fifths of socioeconomic advantage for the Australian population.

**Table 1:** Participant demographics and smoking characteristics (*N* = 124)

	Packs of ≥25 (Analysed, *n* = 39)	Packs of 20 (Analysed, *n* = 40)	Excluded (*n* = 45)
Gender, n (%)			
*Male*	15 (39)	19 (48)	21 (47)
*Female*	24 (62)	21 (52)	24 (53)
Age, mean (SD)	55 (13)	55 (13)	51 (14)
Education level^a^, mean (SD)	8 (3)	8 (3)	8 (3.2)
SEIFA^b^, mean (SD)	3.1 (1.3)	3.1 (1.3)	3 (1.2)
Number of cigarettes smoked per day, n (%)			
*Less than 10*	4 (10)	4 (10)	2 (4)
*11–20*	5 (13)	3 (8)	6 (13)
*21–30*	26 (67)	20 (50)	27 (60)
*More than 30 per day*	4 (10)	13 (33)	10 (22)
Usual pack size, n (%)			
*25*	9 (23)	9 (23)	0 (0)
*26*	2 (5)	4 (10)	9 (20)
*30*	8 (21)	10 (25)	3 (7)
*40*	18 (46)	14 (35)	12 (26)
*50*	2 (5)	2 (5)	20 (43)
*60*^c^	0 (0)	1 (3)	2 (4)
HSI^d^, mean (SD)	2.9 (1.1)	3.1 (1.5)	3.1 (1.2)
MTSS^e^, mean (SD)	2.9 (1.1)	3.1 (1.2)	2.9 (1.3)
AUTOS^f^, mean (SD)	16.6 (7.7)	15.3 (8.0)	15.5 (7.6)

^a^Education Level:1. Some Primary School; 2. Finished Primary School; 3. Some Secondary School; 4. Some Technical Or Commercial/ TAFE; 5. Passed School Certificate / Passed 4th Form / Passed Intermediate / Year 10 Junior or Achievement certificate; 6. Passed 5th Form / Year 11 / Passed Leaving or Sub-senior certificate; 7. Finished Technical School / Commercial College / TAFE (including trade certificate) / other certificate or apprenticeship; 8. Finished or now studying for Matriculation, Higher School Certificate (H.S.C.), V.C.E., Year 12, or Senior Certificate; 9. Some University or some college of Advanced Education training; 10. Diploma from College of Advanced Education or TAFE (Not Degree), Tertiary or Management Training (including Diploma other than University Degree); 11. Now at University or College of Advanced Education; 12. Degree from University or College of Advanced Education; 13. Higher Degree or Higher Diploma (e.g. Ph.D, Masters)

^b^SEIFA (Socio-Economic Indexes for Areas) (lowest 20% of areas given quintile number of 1)

^c^*Note*: Some participants recorded usual pack size as 60; however, this was clarified to be bundles of 2 × 30 packs

^d^HSI = Heaviness of Smoking Index (range 0–6)

^e^MTSS = Motivation to Stop Scale (range 1–7)

^f^AUTOS = Autonomy Over Smoking Scale (range 0–36)

### Sample size re-estimation

The interim analysis took place in February 2019 when 124 participants had been randomised. Due to the need to communicate a decision rapidly to the research agency regarding recruitment, the interim analysis used a dataset without imputations for incomplete information.^1^ Of the 79 participants who finished the study, 61 had complete data available at this stage (28 in control arm, 33 in intervention arm).

The pooled SD of the number of cigarettes smoked per day was 7.4 (Bootstrap 95%CI = 4.8, 9.5). The mean difference in the number of cigarettes smoked between the intervention and control conditions was 1.85 (SD = 7.4, 95%CI = -2.1, 5.8): participants smoked an average of 16.4 cigarettes per day (SD = 6.1) in the control condition, compared to 14.5 cigarettes per day (SD = 8.6) in the intervention condition. The sample size re-estimation indicated that 552 additional participants with complete data would be needed for sufficient power to detect a reduction of two cigarettes per day. To account for attrition, 1122 participants would need to be randomised. This met the pre-specified criterion to terminate the trial due to futility (i.e., more than 250 participants in total would need to be randomised).

The analyses of the data following imputation for incomplete information are reported below (*n* = 79). These changes did not alter the conclusion of the interim analysis to stop the trial.

### Primary outcome: average number of cigarettes smoked per day

#### Primary analysis

Data from participants were included in the study arm to which they were allocated in an intention-to-treat analysis. There was no clear evidence of a difference in the number of cigarettes smoked per day between the study arms (Table 2). Participants in the intervention arm (instructed to smoke from packs of 20 cigarettes) smoked 15.9 cigarettes per day (SD = 8.5) while those in the control arm (instructed to smoke from packs of ≥25 cigarettes) consumed 16.8 cigarettes per day (SD = 6.7). The mean difference was − 0.9 cigarettes per day (95%CI = -4.3, 2.6, SE = 1.7, *t*(77) = − 0.498, *p* = .62, *d* = − 0.11).

**Table 2:** Number of cigarettes smoked per day and measures of smoking behaviour and attitudes (*N* = 79)

	Packs of ≥25 (*n* = 39)	Packs of 20 (*n* = 40)				
	Mean (SD)	Mean (SD)	Mean Difference (Packs of ≥25 minus Packs of 20)	95% CI	*p*	Cohen’s *d*
Number of cigarettes smoked per day						
Primary analysis^a^	16.8 (6.7)	15.9 (8.5)	−0.9	−4.3, 2.6	0.620	− 0.11
Secondary analysis*	16.7 (7.7)	15.9 (7.7)	−0.8	−4.3, 2.6	0.639	−0.25
Per protocol analysis	16.6 (6.7)	16.1 (4.3)	−0.5	−4.2, 3.0	0.766	−0.09
Smoking behaviour and attitudes (post-intervention)**						
HSI^b^	3.1 (1.1)	2.8 (1.1)	−0.30	− 0.82, 0.23	0.265	− 0.26
MTSS^c^	3.8 (1.4)	3.4 (1.4)	−0.46	−1.10, 0.19	0.160	−0.33
AUTOS^d^	17.9 (5.2)	17.6 (5.2)	−0.25	−2.67, 2.16	0.838	−0.05

*adjusted for pack size used at baseline, ** adjusted for same measures taken at baseline

^a^*Due to deviations from normality in the primary and secondary outcomes, all analyses were repeated using a bootstrapping method. These produced similar results*

^b^HSI = Heaviness of Smoking Index (range 0–6)

^c^MTSS = Motivation to Stop Scale (range 1–7)

^d^AUTOS = Autonomy Over Smoking Scale (range 0–36)

#### Secondary analysis

With adjustment for participants’ usual pack size reported at baseline, the estimated means for the number of cigarettes smoked per day for participants in the intervention group and the control group were 15.9 (SD = 7.7) and 16.7 (SD = 7.7) respectively with a mean difference of − 0.8 cigarettes per day (95%CI = − 4.3, 2.6, SE = 1.7). Once again, there was no evidence for a difference between groups (*t*(76) = − 0.471, *p* = .64).

#### Per protocol analysis

Of the 40 participants with complete data allocated to the intervention arm, 25 (63%) adhered to instructions to smoke cigarettes from packs of 20 cigarettes. In the control arm, 38 out of 39 (97%) adhered to instructions to smoke cigarettes from only their usual pack sizes of ≥25 cigarettes.

When only data from the adherent participants (as defined above) were analysed, there was no clear evidence of a difference in the number of cigarettes smoked per day between the study arms (*t*(61) = − 0.299, *p* = 0.77). The mean number of cigarettes smoked by participants in the intervention arm was 16.1 (SD = 4.3) and in the control arm was 16.6 (SD = 6.7) with a mean difference of − 0.5 cigarettes per day (95%CI = -4.2, 3.0).

#### Secondary outcomes

There was no clear evidence of a difference in post-intervention scores of heaviness of smoking (mean difference = − 0.30, *F*(1,72) = 1.263, *p* = 0.27, motivation to stop smoking (mean difference = − 0.46, *F*(1,72) = 2.012, *p* = 0.16) or autonomy over smoking (mean difference = − 0.25, *F*(1,72) = 0.042, *p* = 0.84) between study arms with adjustment for the same measures taken at baseline (Table 2).

## Discussion

The observed difference in the number of cigarettes smoked was in the hypothesised direction, with fewer cigarettes smoked per day in the intervention arm (pack sizes of 20 cigarettes) than the control arm (pack sizes of ≥25 cigarettes). However, the confidence intervals around the mean difference include the possibility of an effect in either direction. Similarly, no differences in heaviness of smoking, motivation to stop smoking or autonomy over smoking were detected.

Larger cigarette packs are being introduced across the global market, prompting calls for the introduction of regulation to cap cigarette pack sizes [4]. To our knowledge, this was the first experimental research to investigate the effect of reducing pack sizes (from ≥25 to 20 cigarettes per pack) on tobacco consumption. The sample size re-estimation, afforded by the adaptive design, indicated that the number of additional participants required to detect the expected effect was not considered feasible within available resources, so the study was terminated at that stage.

### Strengths and limitations

The use of an adaptive design was a strength of the study; ensuring optimal use of resources given a lack of existing experimental evidence regarding an estimate of the effect and an uncertain estimate of the standard deviation of the primary outcome [18]. Terminating the study early prevented researcher time and participant effort from being spent completing a study that would have been underpowered to detect the hypothesised effect.

Several limitations contributed to the early termination of the study. The variability in the primary outcome – number of cigarettes smoked per day – was higher than expected based on our earlier pilot study, leading to a large sample size re-estimation in the interim analysis. There are two possible factors contributing to this higher variability. First, it is possible that the measurement of the number of cigarettes smoked per day was unreliable. We used a novel method of measurement that required participants to label and return their empty cigarette packs. This approach intended to address concern regarding the accuracy of traditional methods; smokers are known to under-report the number of cigarettes they smoke by up to one-third when using survey methods [26]. The labels used in our study required participants to fill in non-mutually exclusive fields. Unfortunately, this sometimes resulted in inconsistent responses that were difficult to interpret (e.g. a label on a pack of 20 cigarettes suggesting that the participant had smoked 20 of these cigarettes themselves and given away five). The full dataset is available (URL to be added if accepted) and includes details of the decisions made regarding interpretation of ambiguous responses (see also Additional File 2).

Second, non-adherence to the intervention may have also increased the variability in the primary outcome. Only 63% of participants in the intervention arm complied with the study procedures to purchase cigarettes in pack sizes of 20, and instead continued to purchase in larger pack sizes more than 10% of the time. This contrasts to the adherence rate of 97% in the control condition. This non-adherence is likely to have undermined the effect of the intervention. Based on feedback from participants after the study, a lack of availability of the correct pack size in participants’ local shops is a possible explanation for the high level of non-adherence. This could be mitigated in a future study by ensuring prior to randomisation that participants can purchase their cigarette packs in their usual pack size (≥25 cigarettes) and a pack size of 20. If more participants had been able to adhere to the intervention, the per-protocol analysis would have carried greater precision in estimating the true impact of the pack size.

Smokers participating in the current study may not be representative of all smokers due to participants self-selecting and needing to be highly motivated to complete the study procedures and to pay closer attention than usual to their smoking which may, inadvertently, have increased their motivation to quit or cut down. Importantly, the current study was designed to detect a difference between randomised groups and so these factors should not cause a bias of the effect estimate as these factors will not affect the separate groups differentially.

The study sample are older than the average population of Australian daily smokers and older smokers generally smoke more and use larger pack sizes. However, the sample was also mostly female and more highly educated than the general population of Australian smokers and use of larger pack sizes is greater among males and the low-SES population [27], who also smoke more cigarettes per day on average [28]. Those with higher levels of consumption and who use larger pack sizes are expected to disproportionately benefit from a capping policy. Therefore, the results of the current study would likely substantially underestimate the magnitude of potential reductions in the total Australian population of smokers, and the potential prevention of escalation in consumption in countries that do not currently have large (> 25) pack sizes.

### Implications for research and policy

Considerable uncertainty remains regarding the true effect of capping cigarette pack sizes at 20 in jurisdictions where this is currently the minimum pack size (i.e., mandating a single pack size of 20 cigarettes only). Given the policy’s possible impact on smoking cessation, further investigation is warranted to produce a reliable effect estimate and establish its potential to contribute to global tobacco control measures [4]. Hoffman and colleagues [29] warn against complacency in tobacco control, highlighting the need for well-implemented, effective policies. These lessons learned from the present study could inform a more efficient RCT. A study with a cross-over design, in which each participant takes part in both study arms, is likely to have greater power to detect an effect because within-person variation in cigarette consumption is generally smaller than between-person variation [30].

In this study we aimed to isolate the impact of pack size from the impact of price by compensating participants for the increase in cost-per-stick they would experience as a result of purchasing cigarettes in a smaller pack size. It is possible that a real-world cap on cigarette pack sizes would help to maintain a high cost per stick by reducing the opportunity for price-related promotions by tobacco companies [4] which may further contribute to the impact of the intervention.

## Conclusion

It remains unclear whether capping cigarette pack sizes at 20 in jurisdictions where this is currently the minimum pack size reduces cigarette consumption. Importantly, the results of this study provides no evidence that capping cigarette pack sizes would be ineffective at reducing smoking. An adaptive design allowed the early termination of a study that would have been underpowered to detect an effect. The limitations identified in this study can inform a more efficient RCT. Given the potential impact of increasing pack sizes on tobacco consumption, and the value of a policy to cap cigarette pack sizes to contribute to reducing global smoking prevalence, further research is urgently required to address the dearth of experimental evidence in this area.

### Supplementary Information

**Additional file 1.** Cigarette pack size and consumption: a pilot randomised controlled trial.

**Additional file 2.** Missing data and inconsistent responses.

## Data Availability

The data that form the basis of the results presented here are available from the University of Cambridge Research Data Repository (https://www.data.cam.ac.uk/repository), DOI: 10.17863/CAM.71656.

## APPENDIX 1

### OSF STUDY DOCUMENTS AND ISRCTN REGISTRATION

The following documents related to this study can be found on the OSF: https://osf.io/g45zn/

01 Jun 2018 ‘Protocol Cig Pack Size_V1.0_31 May 2018.pdf’.

18 Jan 2019 ‘Protocol Cig Pack Size V3.0 03.12.2018.pdf’.

18 Jan 2019 ‘A note on protocol V3.0.pdf’.

11 Jun 2019 ‘Detailed analysis plan for cig pack size_v4 190611.pdf’.

11 Jun 2019 ‘Appendix A. Missing data and inconsistent responses 20190430.pdf’.

In June 2018, we planned to conduct a pilot study, to inform a subsequent RCT, and we registered the protocol on the OSF prior to data collection (Protocol Cig Pack Size_V1.0_31 May 2018.pdf, see https://osf.io/zt8dh for dated registration).

The pilot study had aimed to recruit up to 70 participants in order to estimate the likely standard deviation (SD) of the primary outcome, and the retention rate among those recruited. Owing to a concern that the pool of potential participants within the research agencies panel for the main RCT would be diminished by this external pilot, a decision was made to terminate the pilot study after 17 participants had been randomised and full outcome data had been obtained from 14 participants. We used these data to estimate the within-arm SD. Because the estimate had a wide confidence interval, we opted to begin the main study with an adaptive design (Chow & Chang, 2011). This involves an interim stage of analysis to allow for a more accurate estimation of the SD as a basis for assessing whether the expected effect size can be feasibly demonstrated (A note on protocol V3.0.pdf).

In January 2019, we uploaded a revised RCT protocol (Protocol Cig Pack Size V3.0 03.12.2018′). The RCT, including the hypothesis, was also registered on ISRCTN (10.1186/ISRCTN34202533), after data had been collected but before the interim analysis took place.

In June 2019, a full analysis plan of all outcomes was uploaded prior to conducting data analysis (Detailed analysis plan for cig pack size_v4 190,611.pdf, see https://osf.io/xa7uz for dated registration).

## APPENDIX 2

### INCLUSION AND EXCLUSION CRITERIA

#### Inclusion criteria

i. Aged 18 and over.
ii. Smoke only factory-made cigarettes.
iii. Smoke 5 or more cigarettes a day on every day of the week.
iv. Smoked at least 100 cigarettes in his or her lifetime.
v. Routinely purchase cigarettes in packs of 25 or more.
vi. Use a brand variant in which cigarettes are available in pack sizes of 20 as well as sizes of one or more of the following: 25, 26, 30, 35, 40, 43 and/or 50.
vii. Use a brand that is stocked in a pack size of 20 by at least one of the two major Australian supermarkets in the month before recruitment.
viii. Live anywhere in Australia.
ix. Able to read and write sufficient English to complete all study procedures.
x. Willing to collect and post one week of receipts of cigarettes purchased at baseline.
xi. Willing to record on each cigarette pack dates when the pack was opened and when finished.
xii. Willing to post weekly envelopes – on four consecutive weeks - containing all empty packs of cigarettes smoked in the preceding week with completed forms.
xiii. Willing to undergo a telephone interview at the end of the study.

#### Exclusion criteria

i. Pregnant women.
ii. Intend to quit smoking in the next three months.
iii. Used e-cigarettes weekly over the past month, and intend to continue.
iv. Smoke roll-your-own (RYO) cigarettes.
v. Normally transfer cigarettes into a case.
vi. Don’t usually buy their own cigarettes.
vii. Live in the same household as someone who has enrolled in the study.
viii. Do not own a mobile phone or similar device with the ability to send photos via a text or email message.

## Abbreviations

ISRCTN: International Standard Randomised Controlled Trial Number
RCT: Randomised controlled trial
SD: Standard deviation

## Footnotes

1 In this dataset, dashes written by participants on their labels had been erroneously entered as missing data.
