# Supplementary material for: Cigarette pack size and consumption: an adaptive randomised controlled trial
Source: BMC Public Health. 2021 Jul 18;21:1420. doi: 10.1186/s12889-021-11413-4 (PMC8286601; doi:10.1186/s12889-021-11413-4)
Supplement: Supplementary file 1 — Additional file 1. Cigarette pack size and consumption: a pilot randomised controlled trial. [file 12889_2021_11413_MOESM1_ESM.pdf]

Additional File 1: Cigarette pack size and consumption: a pilot randomised controlled trial

Aims

i. To assess the feasibility and acceptability of a novel method of measuring the number of cigarettes smoked as reflected in the study retention rate;
ii. To measure the standard deviation of number of cigarettes smoked a day to estimate the sample size required for the main study.

Methods

Ethics approval was obtained from the Cambridge Psychology Research Ethics Committee (PRE.2018.037). The study protocol was pre-registered on the Open Science Framework (https://osf.io/g45zn/) before any data were collected.

Study design

In a parallel group randomised controlled trial (RCT), participants (who routinely purchase packs sizes ≥25) were randomised to purchase cigarettes, of the same brand variant which they usually consume, in pack sizes of either: *i.* 20 (intervention arm) or *ii.* 25 or greater (control arm).

Sample and Setting

Eligible Australian smokers (see inclusion and exclusion criteria below) were recruited by a research agency (Roy Morgan https://www.roymorgan.com) from a pre-existing research panel.

The study took place in Australia with participants providing data through telephone calls and mailing back physical forms to the research agency based in Melbourne. Data collection took place between June and August 2018.

Inclusion criteria

i. Aged 18 and over.
ii. Smoke only factory made cigarettes.
iii. Smoke 5 or more cigarettes a day on every day of the week.
iv. Smoked at least 100 cigarettes in his or her lifetime.
v. Routinely purchase cigarettes in packs of 25 or more.
vi. Use a brand or brand variant in which cigarettes are available in pack sizes of 20 as well as sizes of one or more of the following: 25, 26, 30, 35, 40, 43 and/or 50.
vii. Use a brand variant that is stocked in a pack size of 20 by at least one of the two major Australian supermarkets in the month before recruitment.
viii. Live anywhere in Australia.
ix. Able to read and write sufficient English to complete all study procedures.
x. Willing to collect and post one week of receipts of cigarettes purchased at baseline.
xi. Willing to record on each cigarette pack dates when the pack was opened and when finished.
xii. Willing to post weekly envelopes – on four consecutive weeks - containing all empty packs of cigarettes smoked in the preceding week with completed forms.
xiii. Willing to undergo a telephone interview at the end of the study.

Exclusion criteria

i. Pregnant women.
ii. Intend to quit smoking in the next three months.
iii. Used e-cigarettes weekly over the past month, and intend to continue.
iv. Smoke roll-your-own (RYO) cigarettes.
v. Normally transfer cigarettes into a case.
vi. Don't usually buy their own cigarettes.
vii. Live in the same household as someone who has enrolled in the study.
viii. Do not own a mobile phone or similar device with the ability to send photos via a text or email message.

Initially we planned to recruit 134 participants with the expectation of obtaining complete data on 70 participants. This was expected to be sufficient to estimate the pooled standard deviation of the number of cigarettes smoked a day (1,2) and the retention rate with reasonable precision.

Participants were reimbursed up to AUD$240 ($15 for completing the baseline, $50 for week 1, $35 for each of weeks 2 – 4 and $70 for completing the debrief interview) for taking part in the study. Participants who did not complete all study weeks but agreed to take part in a debrief interview were reimbursed $20 for the interview. Participants allocated to the intervention were reimbursed for the average cost incurred as a result of purchasing cigarettes in smaller pack sizes.

Randomisation

A simple randomisation sequence was generated by a senior statistician (RM) not involved with recruitment or data collection. The random number list was given to the research agency. Participants' allocation to condition was concealed until after consent and completion of the baseline phase (see below). Participants were blinded to the study hypothesis but were aware that they may be asked to purchase cigarette packs in different sizes. The analyst completing the data analysis (KDL) was blinded to allocation.

Materials

Cigarette pack labels

A set of white, green and red adhesive labels (5.5 x 6.5cm) were provided to participants for attaching to their cigarette packs to record information that would allow calculation of how many cigarettes they had smoked. White labels had space for participants to fill in the following information: date started, date finished, number of cigarettes they smoked from the pack, number they gave away and the number of cigarettes smoked from another pack during the stated dates. The green labels were for cigarette packs that participants already had open at the beginning of the study and additionally had space for participants to fill in how many cigarettes were in the pack at the start of the study. The red labels were for cigarette packs open at the end of the study and contained an additional field for the number of cigarettes remaining in the pack at the end of the study.

Study packs

A study pack was mailed to each participant which contained written instructions, a set of labels, return slips and the pre-paid return envelopes participants would use to mail back their finished cigarette packs at the end of each week to the research agency.

Measures

Primary outcomes

i. *Feasibility and acceptability*

A pre-specified criterion for the feasibility of a full RCT was that at least 70% of randomised participants provide data for the planned primary outcome - number of cigarettes smoked a day. The feasibility of recruitment and implementation of the study protocol were also taken into consideration.

Acceptability of the study procedures was interpreted qualitatively from debrief interviews with participants.

ii. *Standard deviation of number of cigarettes smoked*

The number of cigarettes smoked each day was calculated from the labelled empty cigarette packs participants returned at the end of each of the four weeks of the study. Requiring participants to provide their empty cigarette packs allowed their compliance with the intervention to be checked and made it less likely that participants would forget to report the cigarettes they had smoked during the four week study period.

The total number of cigarettes participants smoked across the four weeks of the study was divided by 28 to obtain a measure of daily consumption. The pooled standard deviation between the study arms was calculated and used in the sample size calculation for the main study.

Additional measures

a. *Demographic characteristics*

Age and gender were recorded at recruitment. Socioeconomic status was measured using the Australian index of Relative Socio-Economic Advantage and Disadvantage (SEIFA, 3). This reflects a combination of education level, income and occupation of respondent. Scores (out of 60) for each of these three categories were combined and summarised in quintiles ranging from the first quintile with the lowest socioeconomic status to the fifth with the highest.

b. *Smoking characteristics*

Three aspects of smoking behaviour were completed over the telephone at recruitment and at the end of the study:

i. Heaviness of Smoking Index (HSI): a two-item measure of the number of cigarettes smoked per day and time to first cigarette (4).

ii. Motivation to Stop Scale (MTSS): a single-item measure (5) with responses to the question: Which of the following describes you? Responses range from 1, I don’t want to stop smoking to 7, I REALLY want to stop smoking and intend to in the next month.

iii. Autonomy Over Smoking Scale (AUTOS): a 12-item measure of tobacco dependence (6,7).

Procedure

To avoid participants focusing on their smoking in relation to pack size, the study was presented as investigating how cigarette pack size affects the perception of health warnings. The study aims were revealed to participants as they ended their participation in a funnel-debrief.

After providing written informed consent, participants took part in a baseline week which would familiarise them with what would be required during the study weeks. Participants received one express pre-paid envelope in which to return date-stamped receipts for cigarette packs purchased over the forthcoming week.

Participants who successfully completed the baseline week were then randomly allocated to one of the two study arms and sent a study pack by post. For four weeks, participants were instructed to smoke their regular brand variant of cigarettes from only the pack size appropriate to their allocated study arm (20 for participants in the intervention arm, ≥25 for the control arm). Each week, participants attached the provided adhesive labels to all of the cigarette packs they finished, filled in the required information and mailed them back to the research agency at the end of the week.

Text and telephone call reminders were sent to participants during the study to maximise fidelity to the protocol.

A telephone interview was conducted within two weeks of the receipt of the final envelope. The interview included a funnel debrief of study procedures. Participants who did not comply with study procedures and those who did not complete all weeks of the study were also invited to take part in the interview to find out about their experience of taking part and why they withdrew.

Data analysis

Analysis was conducted by an analyst (KDL) not involved in the collection of the data and blinded to allocation. Descriptive statistics were calculated for the baseline characteristics of participants in the two study arms. IBM SPSS 24 software was used for data analysis. The recruitment rate and standard deviation are reported alongside 95% confidence intervals (CI).

Results

Sample characteristics

Following concern from the research agency that the pool of eligible participants was smaller than anticipated and would be exhausted by the pilot study, the pilot study was stopped early. A total of 23 participants were recruited to the baseline week and 17 successfully completed the study requirements and were randomised to one of the study arms. The study CONSORT flow diagram is provided in Figure 1.

[Insert Figure 1]

Mean age of the 17 randomised participants was 47.1 (SD = 12.1). Participants were mostly women (16 women, 1 man), had a median education level of 7.0 (IQR = 3.0-10.0), and a mean socioeconomic quintile of 2.6 with 17.6% of participants in the top two fifths of

socioeconomic advantage for the Australian population. See Table 1 for complete demographic information and smoking characteristics.

[Insert Table 1]

<u>*Primary outcomes*</u>

Feasibility and acceptability

A large proportion of those assessed for eligibility did not meet the inclusion criteria (89.3%, 192/215). The recruitment rate from the pool of eligible participants was 57.5% (23/40).

The retention rate was 14 out of the 17 participants who were randomised [82.4% (95% CI = 56.6, 96.2) providing complete data for the number of cigarettes smoked per day.

The debrief interview

Of the 17 randomised participants, 7 (41%) took part in the debrief interview. Responses indicated that participants believed the study had been about warning labels, as per the cover story. No one objected to being deceived in this way and most participants found the procedures easy to follow and agreed it was acceptable to ask people to purchase cigarettes in differently sized packs for the purposes of research. One participant who had been allocated to the intervention and subsequently dropped out of the study felt it was a financial burden to switch from cigarette packs of 40 to 20 because of the greater value for money that the larger packs provide, despite the compensation offered for this.

Secondary outcome

The pooled within-group SD of the number of cigarettes smoked per day was 5.1 (95% CI = 3.7, 8.2). Participants in the control arm (smoking from pack sizes of 25 cigarettes or greater) consumed 16.5 cigarettes per day (SD = 5.0) while those in the intervention arm (smoking from pack sizes of 20) smoked 14.6 cigarettes per day (SD = 5.4). The mean difference was -1.9 cigarettes per day (95% CI = -7.9, 4.2).

Discussion

The results of the pilot indicated that an RCT to assess the impact on smoking of capping cigarette pack sizes at 20 was feasible and acceptable.

The recruitment rate was lower than anticipated with a large proportion of the people screened not being eligible for the study. This was predominantly due to the requirement that participants smoke only factory-made cigarettes and smoke at least five cigarettes a day. Substantial dropout (26%) occurred once participants were recruited to the baseline week. Changes to the reimbursement schedule were made in attempt to ameliorate this drop out for the main study. The retention rate of randomised participants contributing data to the primary outcome (82%) exceeded the pre-specified criterion (70%) considered to make recruitment to an RCT feasible.

Owing to the small number of participants, the estimated standard deviation of the number of cigarettes smoked was relatively imprecise with large confidence intervals. An RCT with an adaptive design was used in the main study to allow for a more precise estimate of the standard deviation at an interim stage of data collection and re-estimate the sample size

required to detect a reduction of 2 cigarettes per day. Adaptive designs are valuable for making decisions concerning the expected value to be gained from further data collection (8).

References

1. Teare M, Dimairo M, Shephard N, Hayman A, Whitehead A, Walters SJ. Sample size requirements to estimate key design parameters from external pilot randomised controlled trials: a simulation study. Trials. 2014;15(1):264.

2. Whitehead AL, Julious SA, Cooper CL, Campbell MJ. Estimating the sample size for a pilot randomised trial to minimise the overall trial sample size for the external pilot and main trial for a continuous outcome variable. Stat Methods Med Res. 2016;25(3):1057–73.

3. Census of Population and Housing: Socio-Economic Indexes for Areas (SEIFA), Australia, 2011 (cat. no. 2033.0.55.001).

4. Heatherton TF, Kozolowski LT, Frecker RC, Rickert W, Robinson J. Measuring the Heaviness of Smoking: using self-reported time to the first cigarette of the day and number of cigarettes smoked per day. Br J Addict. 1989;84(7):791–800.

5. Kotz D, Brown J, West R. Predictive validity of the Motivation To Stop Scale (MTSS): A single-item measure of motivation to stop smoking. Drug Alcohol Depend. 2013;128(1–2):15–9.

6. DiFranza JR, Wellman RJ, Ursprung WWSA, Sabiston C. The Autonomy Over Smoking Scale. Psychol Addict Behav. 2009;23(4):656–65.

7. DiFranza JR, Savageau JA, Wellman RJ. A comparison of the Autonomy over Tobacco Scale and the Fagerström Test for Nicotine Dependence. Addict Behav. 2012;37(7):856–61.

8. Kumar A, Chakraborty B. Interim analysis: A rational approach of decision making in clinical trial. J Adv Pharm Technol Res. 2016;7(4):118.

Figures and tables

Figure 1. CONSORT flow diagram for the pilot study.

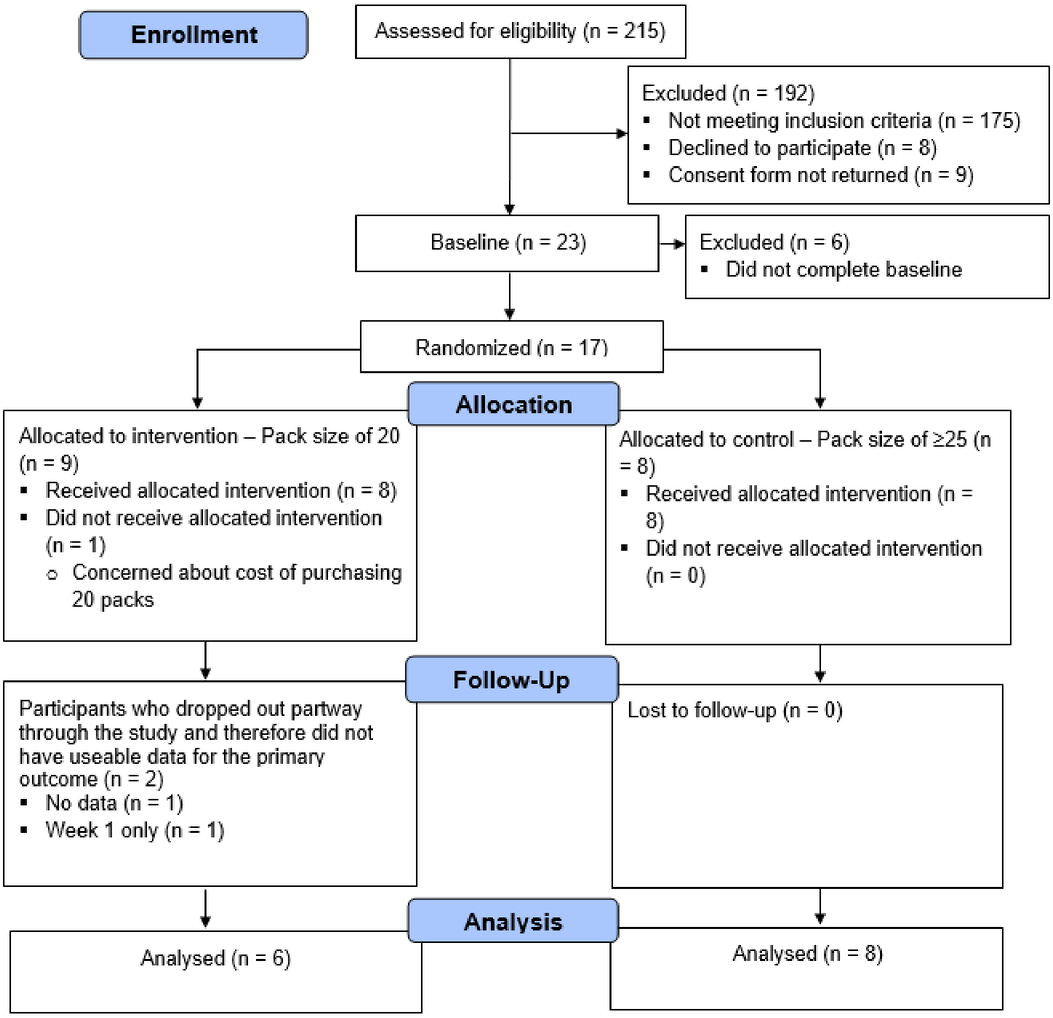

Table 1. Participant demographics, smoking characteristics at baseline and number of cigarettes smoked

	Packs of ≥25 (n = 8)	Packs of 20 (n = 9)
**Demographics**		
Gender, n (%)		
Male	1 (13)	0 (0)
Female	7 (87)	9 (100)
Age, mean (SD)	49 (12)	46 (12)
Education level[a], mean (SD)	8 (3)	7 (4)
SEIFA[b], mean (SD)	2.4 (1.4)	2.8 (1.2)
**Smoking characteristics at baseline**		
Number of cigarettes smoked per day, n (%)		
Less than 10		
11-20	1 (13)	2 (22)
21-30	5 (63)	7 (78)
More than 30 per day	2 (25)	0
	0	0
Usual pack size, n (%)		
25	0 (0)	2 (22)
26	0 (0)	1 (11)
30	2 (25)	1 (11)
40	5 (63)	5 (57)
50	0 (0)	0 (0)
60	1 (13)	0 (0)
HSI[c], mean (SD)	3.3 (1.2)	2.2 (1.1)
MTSS[d], mean (SD)	2.0 (0)	3.1 (1.2)
AUTOS[e], mean (SD)	19.4 (9.2)	15.9 (5.3)
**Number of cigarettes smoked per day**, mean (SD)	16.5 (5)	14.6 (5.4)

Variable key

a) Education Level

1. Some Primary School
2. Finished Primary School
3. Some Secondary School
4. Some Technical Or Commercial/ TAFE
5. Passed School Certificate / Passed 4th Form / Passed Intermediate / Year 10 Junior or Achievement certificate
6. Passed 5th Form / Year 11 / Passed Leaving or Sub-senior certificate
7. Finished Technical School / Commercial College / TAFE (including trade certificate) / other certificate or apprenticeship
8. Finished or now studying for Matriculation, Higher School Certificate (H.S.C.), V.C.E., Year 12, or Senior Certificate
9. Some University or some college of Advanced Education training
10. Diploma from College of Advanced Education or TAFE (Not Degree), Tertiary or Management Training (including Diploma other than University Degree)

11. Now at University or College of Advanced Education
12. Degree from University or College of Advanced Education
13. Higher Degree or Higher Diploma (e.g. Ph.D, Masters)

b) SEIFA (Socio-Economic Indexes for Areas) (lowest 20% of areas given quintile number of 1).
c) HSI = Heaviness of Smoking Index (range 0 - 6)
d) MTSS = Motivation to Stop Scale (range 1 - 7)
e) AUTOS = Autonomy Over Smoking Scale (range 0–36)
