# Supplementary material for: Cigarette pack size and consumption: an adaptive randomised controlled trial
Source: BMC Public Health. 2021 Jul 18;21:1420. doi: 10.1186/s12889-021-11413-4 (PMC8286601; doi:10.1186/s12889-021-11413-4)
Supplement: Supplementary file 2 — Additional file 2. Missing data and inconsistent responses. [file 12889_2021_11413_MOESM2_ESM.pdf]

Additional File 2: Missing data and inconsistent responses

Calculation of primary outcome

The primary outcome is the average number of cigarettes smoked per day during the four weeks of the study (*o*). Participants sent their labelled cigarette packs to the research agency at the end of each week. The number of cigarettes smoked from each pack (*p*) was calculated using the information participants filled in on the labels (described below). The total number of cigarettes smoked over the four-week study period (*t*) was calculated by summing across cigarette packs ($t = \Sigma p$). For each participant, the total number of cigarettes smoked over the four-week study period was divided by 28 to obtain the primary outcome ($o = t/28$)

The labels

There were three different coloured sticky labels with information to fill in:

- Green: for packs that were already open at the beginning of the study period
- Red: packs that were open but not finished at the end of the study period
- White: for all other packs (See Figure 1 for an example)

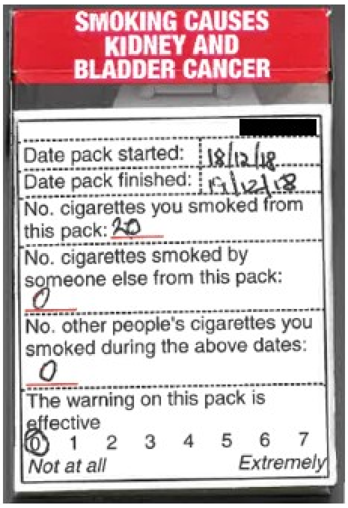

Figure 1. Example of a completed label

Calculating number of cigarettes smoked from each pack (*p*)

To calculate the number of cigarettes participants smoked from each cigarette pack (*p*), the information participants filled in on each label were used (variables *a* – *f*, listed in Table 1).

The formula for calculating the number of cigarettes smoked from each pack, *p* is: $p = b + d$.

The variables a, c, e and f are used to verify participants’ responses.

Missing data

It was agreed by the study team that missing data for the variables required to calculate *p* would be handled as described in Table 2 for the primary and sensitivity analyses. Results of the sensitivity analysis were not different to the primary analysis. Participants in the intervention arm (instructed to smoke from packs of 20 cigarettes) smoked 15.9 cigarettes per day (SD=8.5) while those in the control arm (instructed to smoke from packs of 25 or more

cigarettes) consumed 16.8 cigarettes per day (SD=6.6). The mean difference was -0.9 cigarettes per day (95%CI=-4.3, 2.6, SE=1.7, $t(77)=-0.499$, $p=.62$, $d=-0.11$).

Table 1. Variables used to calculate the primary endpoint and imputations for missing data

		**Imputed values if missing**	
**Variable**	**Description**	**Primary Analysis**	**Sensitivity Analysis**
a	Size of cigarette pack	Mean for that participant	Mean for that participant
b	Number of cigarettes smoked by participant from this pack	Mean for that participant	Pack Size
c	Number of cigarettes given to someone else from this pack	Mean for that participant	Zero
d	Number of cigarettes smoked from another pack during stated dates	Zero	Zero
e	Number of cigarettes in the pack on Monday morning (GREEN STICKERS ONLY)	Full pack size	Full pack size
f	Number of cigarettes remaining in the pack at the end of week 4 (RED STICKERS ONLY)	Zero	Zero

Inconsistent responses

The responses of some participants to the questions on the labels are not internally consistent (e.g. they indicate that they have smoked 22 cigarettes and given away 6 cigarettes from a pack of 25). The method to identify inconsistent responses on white, green and red stickers are described below along with agreed methods for correcting these responses.

All labels

The size of the cigarette pack should not be less than the number of cigarettes smoked by the participant plus the number of cigarettes they gave to someone else. The formula for identifying consistent responses is:

If $a \geq b + c$ then the responses are consistent

There were occasions when the number of cigarettes participants say they have smoked from a pack (b) plus the number they gave away (c) was greater than the size of the cigarette pack (a). We only use variables b and d to calculate the primary endpoint so we assume that their answer to b is correct.

Green stickers

The number of cigarettes in the pack on Monday morning should be greater than or equal to the number of cigarettes they smoked from the pack plus the number they gave to someone else (neglecting pack size in this equation?) The formula for identifying consistent responses is:

If $e \geq b + c$ then responses are consistent

Example of inconsistent responses:

a) Size of cigarette pack - 25
b) Number of cigarettes smoked by participant from this pack - 20
c) Number of cigarettes given to someone else from this pack - 5
d) Number of cigarettes smoked from another pack during stated dates - 0
e) Number of cigarettes in the pack on Monday morning – 10

A common participant error was to fill in the green stickers with information relating to the whole pack of cigarettes rather than just the cigarettes that were in the pack on Monday. Therefore we don’t know how many cigarettes were smoked before or during the study. We have so far imputed the number of cigarettes smoked from the pack (b*) as:

$b^* = (b/(b+c))^*e$

Then calculate the number of cigarettes smoked as $p = b^* + d$

Red stickers

Response consistency check for red stickers: the number of cigarettes used before the end of the study (the pack size minus the number of cigarettes remaining in the pack) should be greater than or equal to the number of cigarettes smoked by the participant plus the number they gave to someone else. The formula for identifying consistent responses is:

If $(a - f) \geq b + d$, the responses are consistent

A common participant error was to fill in the red stickers with information relating to the whole pack of cigarettes rather than just the cigarettes that they consumed before the end of the study. Therefore we don’t know how many cigarettes were smoked during or after the study. We imputed the number of cigarettes smoked (b^*) from the pack as follows:

$b^* = (b/(b+c))^*(a-f)$

Then calculate the number of cigarettes smoked as $p = b^* + d$

‘Cigarette pack size and consumption: an adaptive randomised controlled trial’
